# Mucosal associated invariant T cells from human breast ducts mediate a Th17-skewed response to bacterially exposed breast carcinoma cells

**DOI:** 10.1186/s13058-018-1036-5

**Published:** 2018-09-12

**Authors:** Nicholas A. Zumwalde, Jill D. Haag, Michael N. Gould, Jenny E. Gumperz

**Affiliations:** 1 Department of Medical Microbiology and Immunology, University of Wisconsin School of Medicine and Public Health, Madison, WI USA; 2 McArdle Laboratory for Cancer Research, Department of Oncology, University of Wisconsin School of Medicine and Public Health, Madison, WI USA

**Keywords:** Mucosal associated invariant T (MAIT) cells, Breast duct, Breast carcinoma, Microbe-reactive human T cells, NKG2D ligands, Th17 response

## Abstract

**Background:**

Antimicrobial T cells play key roles in the disease progression of cancers arising in mucosal epithelial tissues, such as the colon. However, little is known about microbe-reactive T cells within human breast ducts and whether these impact breast carcinogenesis.

**Methods:**

Epithelial ducts were isolated from primary human breast tissue samples, and the associated T lymphocytes were characterized using flow cytometric analysis. Functional assays were performed to determine T-cell cytokine secretion in response to bacterially treated human breast carcinoma cells.

**Results:**

We show that human breast epithelial ducts contain mucosal associated invariant T (MAIT) cells, an innate T-cell population that recognizes specific bacterial metabolites presented by nonclassical MR1 antigen-presenting molecules. The MAIT cell population from breast ducts resembled that of peripheral blood in its innate lymphocyte phenotype (i.e., CD161, PLZF, and interleukin [IL]-18 receptor coexpression), but the breast duct MAIT cell population had a distinct T-cell receptor Vβ use profile and was markedly enriched for IL-17-producing cells compared with blood MAIT cells. Breast carcinoma cells that had been exposed to *Escherichia coli* activated MAIT cells in an MR1-dependent manner. However, whereas phorbol 12-myristate 13-acetate/ionomycin stimulation induced the production of both interferon-γ and IL-17 by breast duct MAIT cells, bacterially exposed breast carcinoma cells elicited a strongly IL-17-biased response. Breast carcinoma cells also showed upregulated expression of natural killer group 2 member D (NKG2D) ligands compared with primary breast epithelial cells, and the NKG2D receptor contributed to MAIT cell activation by the carcinoma cells.

**Conclusions:**

These results demonstrate that MAIT cells from human breast ducts mediate a selective T-helper 17 cell response to human breast carcinoma cells that were exposed to *E. coli*. Thus, cues from the breast microbiome and the expression of stress-associated ligands by neoplastic breast duct epithelial cells may shape MAIT cell responses during breast carcinogenesis.

**Electronic supplementary material:**

The online version of this article (10.1186/s13058-018-1036-5) contains supplementary material, which is available to authorized users.

## Background

Most breast cancers initially arise in the epithelial ducts [1]. Although epithelial surfaces of the gastrointestinal and urogenital tracts are well known to be colonized by intricate microbial communities, it has only recently become clear that the breast ducts also contain a complex microbiota [2–8]. Because T cells that are specific for microbial antigens are now known to play key roles in the progression of tumors arising in epithelial layers of the intestine [9], the observation that the breast ducts contain microbial colonists raises the question whether antimicrobial T cells may also contribute, either positively or negatively, to the genesis of breast cancers. Consistent with this possibility, the presence of cancerous tissue has been found to be associated with alterations to the microbiome of the local breast tissue [8]. Hence, dysbiosis of the breast duct microbiome might lead to increased or altered T-cell activation. A central obstacle to assessing the role of microbe-specific T cells in breast cancer is that little is known about the T-cell compartment found within human breast ducts, and particularly, the presence of T cells that recognize microbial antigens has not yet been established.

We recently investigated the intraepithelial lymphocyte (IEL) compartment from isolated human breast epithelial duct organoids and observed that it includes T cells with Vα7.2^+^ T-cell receptors (TCRs) [10]. TCR use of the Vα7.2 segment (T-cell receptor alpha variables 1 and 2 [TRAV1–2]) is one of the central characteristics of a distinctive subset called *mucosal associated invariant T cells* (MAIT cells) [11]. MAIT cells are innate T cells that recognize specific microbially synthesized precursors of riboflavin as antigens presented by the nonclassical antigen-presenting molecule MR1 [12, 13] and are thus microbially reactive T cells. They typically coexpress CD161, promyelocytic leukemia zinc finger protein (PLZF), and interleukin (IL)-18Rα and can be readily detected using MR1 tetramers loaded with 5-(2-oxoprophylideneamino)-6-d-ribitylaminouracil (5RU) [12, 14–16]. MAIT cells are comparatively abundant in human peripheral blood, typically comprising 0.5–10% of the T-cell population [16]. MAIT cells have also been detected in a variety of other tissues, including liver, lung, kidney, intestine, female genital tract, prostate, and ovary [14, 17–22]. MAIT cells from blood mainly produce interferon (IFN)-γ and tumor necrosis factor (TNF)-α upon activation, and they efficiently mediate cytolytic responses [23]. In contrast, compared with those from the blood, MAIT cells from the female genital tract expressed higher levels of T-helper 17 cell (Th17) cytokines (IL-17A and IL-22) and lower levels of Th1 cytokines (IFN-γ and TNF-α) in response to *Escherichia coli* [20]. Thus, MAIT cells from distinct anatomical locations may have important functional differences.

Intriguingly, recent studies suggest that MAIT cells may play a role in the etiology of colon adenocarcinomas. MAIT cells were found to accumulate at tumor sites in patients with colon cancer, and the tumor-associated MAIT cells produced lower levels of IFN-γ than those obtained from healthy intestinal tissue from the same donor [24]. In another study, circulating MAIT cells from patients with colorectal cancer were found to have reduced expression of IFN-γ and TNF-α and elevated levels of IL-17A compared with MAIT cells from the blood of healthy control subjects [25]. It is not yet clear whether the apparent Th17 bias of tumor-associated and blood MAIT cells observed in patients with colon cancer is due to a functional skewing that occurs in the context of malignancy or whether it is a result of the expansion of a MAIT cell subset that is normally present only within select mucosal epithelial sites. Similarly, the role of microbial stimulation and/or dysbiosis in the MAIT cell response during colon cancer is as yet unknown. Nevertheless, the observation that Th17-biased MAIT cells are recruited to the sites of colon adenocarcinomas raises the possibility that these T cells also play a role in breast carcinomas. Therefore, in this analysis, we sought to investigate the phenotypes and functional characteristics of breast epithelium-derived MAIT cells, as well as to determine the ability of microbially exposed breast carcinoma cells to elicit responses from human MAIT cells.

## Methods

### Breast tissue acquisition and preparation

Noncancerous breast tissue from reduction mammoplasties or prophylactic mastectomies was obtained from the Cooperative Human Tissue Network (a National Cancer Institute-supported resource) or from the UW Translational Science BioCore-BioBank, in accordance with an institutional review board (IRB)-approved protocol. Human breast epithelial organoids were isolated as previously described [10]. Briefly, breast tissue was minced and digested overnight in a 37 °C shaker with 1× collagenase/hyaluronidase in Complete EpiCult B Human Media (STEMCELL Technologies, Vancouver, BC, Canada) supplemented with 5% FBS (HyClone; GE Healthcare Bio-Sciences, Pittsburgh, PA, USA). After incubation, digested tissue was spun for ≤ 1 minute at 80–100 × *g* to produce a pellet enriched for epithelial ductal organoids. The pellet was washed, and organoids were collected on a 40-μm filter. Organoids were cryopreserved in 50% FBS/6% dimethyl sulfoxide and stored in liquid nitrogen. Single-cell suspensions from organoids were prepared for all experiments by trypsinizing the organoids using 2-3 ml of 0.1% ethylenediaminetetraacetic acid (EDTA)/trypsin solution (diluted in PBS from 0.5%; Life Technologies, Carlsbad, CA, USA) for ≥ 3 minutes. EDTA/trypsin reaction was quenched using serum-containing media and spun. Pellets were resuspended and filtered using 40–70-μm filters. Cells were spun, supernatants discarded, and breast organoid-derived cells resuspended for experimentation.

### Peripheral blood mononuclear cell isolation

Peripheral blood mononuclear cells (PBMCs) were isolated from healthy donors according to an IRB-approved protocol. Written informed consent was obtained from all donors. PBMCs were isolated from blood using Ficoll-Paque PLUS (GE Healthcare Bio-Sciences) as previously described [10].

### Flow cytometric analyses

For surface stains, cells were washed with PBS, blocked with 20% human AB serum (Atlanta Biologicals, Flowery Branch, GA, USA) for 15 minutes, stained with fluorochrome-conjugated antibodies for 30 minutes at 4 °C, washed, resuspended in PBS, and analyzed by flow cytometry (BD LSR II cytometer; BD Biosciences, San Jose, CA, USA) with FlowJo analysis software (version 9.3.1; FlowJo, Ashland, OR, USA). MR1 tetramers (5RU and 6FP provided by National Institutes of Health, Bethesda, MD, USA) were used at a 1:100 final dilution. In general, optimal staining was seen when tetramers were stained for 40 minutes in the dark at room temperature prior to surface staining. Intracellular cytokine staining was performed according to the manufacturer’s recommendations using the BD Cytofix/Cytoperm kit in the presence of BD GolgiStop or BD GolgiPlug protein transport inhibitors (BD Biosciences). PLZF staining was performed with the BD Cytofix/Cytoperm kit.

The following fluorochrome-conjugated flow cytometry antibodies were used for analysis: CD45 (clone HI30), CD3 (OKT3), Vα7.2 (3C10), CD161 (HP-3G10), IL-18Rα (H44), TNF-α (Mab11), IFN-γ (4S.B3), IL-17A (BL168), natural killer group 2 member D (NKG2D) (1D11), CD31 (WM59), epithelial cell adhesion molecule (9C4), CD49f (GoH3), major histocompatibility complex class I-related chains A and B (MICA/B) (6D4), CD56 (HCD56), MR1 (26.5), and NKp46 (9E2) (all from BioLegend, San Diego, CA, USA); Vβ2 (MPB2D5), Vβ8 (56C5.2), Vβ13.1 (IMMU 222), Vβ13.2 (H132), and Vβ13.6 (JU74.3) (all from Beckman Coulter Life Sciences, Indianapolis, IN, USA); UL16-binding protein 1 (ULBP1) (170818), ULBP2/5/6 (165903), ULBP3 (166510), ULBP4 (709116), immunoglobulin G2A (IgG2A) (20102), and IgG2B (133303) (R&D Systems, Minneapolis, MN, USA); and PLZF (R17-809; BD Pharmingen, San Diego, CA, USA).

### Short-term in vitro expansion of MAIT cells

Single-cell suspensions prepared from breast duct organoids or freshly isolated PBMCs were stained using 5RU-loaded MR1 tetramer and antibodies against CD45, CD3, Vα7.2 TCR, and CD161. MAIT cells (1–1000 cells/well) were sorted into 96-well round-bottomed plates. Irradiated PBMCs were added at a density of 1 × 10^5^ cells/well in T-cell medium (RPMI 1640, 15% heat-inactivated bovine calf serum, 3% human AB serum, 1% penicillin/streptomycin [P/S], 200 U/ml recombinant human IL-2) containing 5 μg/ml phytohemagglutinin (PHA; Sigma-Aldrich, St. Louis, MO, USA), and the cultures were maintained at 37 °C in a humidified incubator with 5% CO_2_. If necessary, the cells were restimulated after 4–6 weeks to induce another round of proliferation by adding irradiated PBMCs in T-cell medium containing 5 μg/ml PHA. The MAIT cell composition of the expanded cells was assessed by flow cytometry after ~ 8 weeks of in vitro expansion using 5RU-loaded MR1 tetramer staining, and lines that were comprised of ≥ 95% MAIT cells were used for functional analyses.

### Phorbol 12-myristate 13-acetate and ionomycin stimulation

Cells were washed and resuspended in culture medium (RPMI 1640, 15% HI-BCS, 3% human AB serum, 1% P/S) containing a final concentration of 50 ng/ml phorbol 12-myristate 13-acetate (PMA) (Sigma-Aldrich) and 500 ng/ml ionomycin (Sigma-Aldrich) in the presence of monensin or brefeldin A (BD Biosciences). The cells were stimulated for ~ 6 hours at 37 °C. Unstimulated control cells were incubated in parallel in culture medium alone. Because we observed that effector cytokine production decreased as cell density increased (data not shown), we used a maximum density of 2.5 × 10^5^ cells/well for stimulation. After stimulation, cells were harvested and prepared for flow cytometry.

### Breast carcinoma cells and bacterial exposure

The breast carcinoma cell line MDA-MB-231 was obtained from the American Type Culture Collection (Manassas, VA, USA) as an authenticated cell line and maintained in DMEM/F-12 medium (Corning, Corning, NY, USA) supplemented with 10% HI-BCS or 10% FBS and 1% P/S; Mediatech, Manassas, VA, USA). To prepare carcinoma cells for functional assays, the carcinoma cells were plated at a subconfluent density in flat-bottomed tissue culture plates and allowed to adhere for ~ 3 hours at 37 °C. *E. coli* strain K12 was cultured in Luria-Bertani (LB) broth in a 37 °C shaker overnight and then stored frozen in 40% glycerol/LB broth. Aliquots were thawed prior to use, and *E. coli* was washed in PBS three times. Bacteria were fixed in 1% formalin for 3 minutes, then washed three times with PBS and resuspended in the DMEM/F-12 medium used to culture the tumor cells. Carcinoma cells were exposed to *E. coli* overnight at a multiplicity of infection of 400–500 or incubated in medium alone (mock). The wells were then washed with PBS, and fresh DMEM culture medium containing 10% serum and 1% P/S was added.

### Enrichment of primary MAIT cells from PBMCs

We performed a magnetic sorting step to remove potential MR1^+^ antigen-presenting cell (APC) types (e.g., monocytes, B cells, dendritic cells) from PBMC samples prior to using them to test the responses of primary MAIT cells to MDA-MB-231 cells as APCs. CD161^+^ cells were positively selected using indirect magnetic bead separation (Miltenyi Biotec, Bergisch Gladbach, Germany). The PBMCs were incubated first with an anti-CD161-phycoerythrin (PE) antibody and then with anti-PE microbeads, followed by passage over a Miltenyi Biotec LS column. Purity of the resulting cell preparations was assessed by flow cytometric analysis, as shown in Additional file 1: Figure S4.

### Functional assays

Cell preparations containing primary MAIT cells (CD161^+^ PBMCs or breast epithelial organoid cells) or in vitro-expanded MAIT cultures were added to wells containing *E. coli*-exposed or mock-treated MDA-MB-231 breast carcinoma cells. CD161^+^ PBMCs and in vitro-expanded MAIT cells were added at a 1:1 ratio to breast carcinoma cells, whereas the breast epithelial organoid cells (which are composed of both IELs and epithelial cells) were added at a 3:1 ratio. Where indicated, the following blocking antibodies were added to the cocultures: 20 μg/ml anti-MR1 (clone 26.5; BioLegend) or 5 μg/ml anti-NKG2D (1D11; BioLegend). The carcinoma and effector cells were coincubated at 37 °C for ~ 18 hours, then monensin (GolgiStop) or brefeldin A (GolgiPlug) was added to all cultures, and the cells were coincubated for an additional 6 hours. After ~ 24 hours of coincubation, the effector cells were resuspended using cold EDTA (500 mM) in PBS, washed, and analyzed by flow cytometry.

### Statistical analysis

To assess statistical significance, samples from different tissues (e.g., PBMC vs. breast duct organoids) were analyzed using a Mann-Whitney *U* test. Different populations of cells within the same sample (e.g., MAIT cells vs. non-MAIT cells from breast duct organoid preparations) were evaluated using a Wilcoxon matched pairs analysis. Where indicated, a two-tailed, one-sample *t* test was used to assess whether individual treatment groups showed a significant difference compared with a hypothetical value of 100%.

## Results

### Identification of MAIT cells in human breast epithelium

**Fig. 1:**
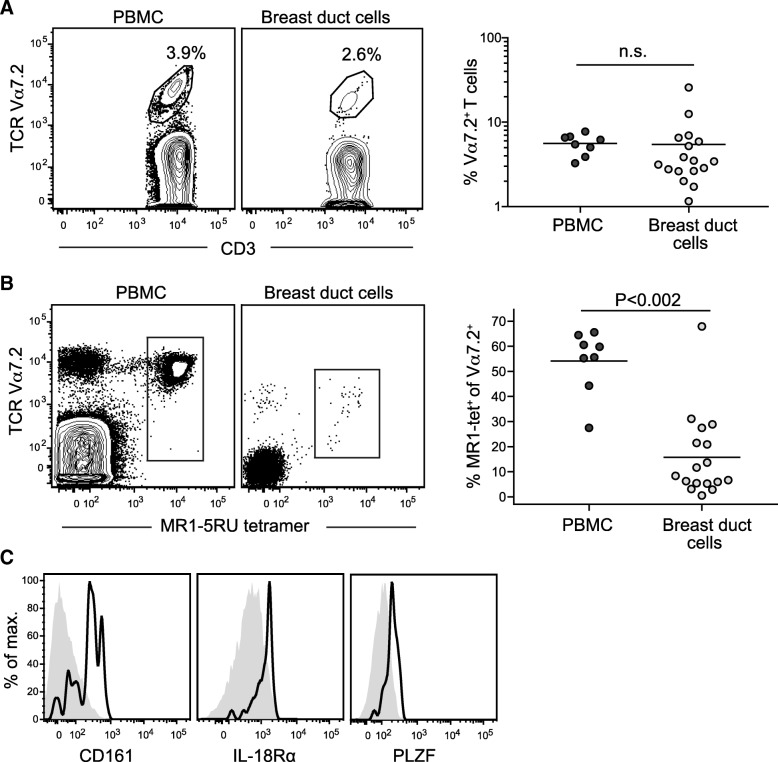
Detection of mucosal associated invariant T cells from human breast ducts. **a** Left plots: Staining for Vα7.2 T-cell receptor TCR expression by T cells from peripheral blood (peripheral blood mononuclear cells [PBMCs]) or human breast ducts. Right plot: Quantification of Vα7.2^+^ T-cell frequencies in PBMCs vs. breast duct tissue. PBMCs and breast tissue samples were not obtained from the same donors. **b** Left plots: Costaining of CD3^+^ cells from blood vs. breast ducts by anti-Vα7.2 antibody and 5-(2-oxoprophylideneamino)-6-d-ribitylaminouracil (5RU)-loaded MR1 tetramer. Right plot: Quantification of the fraction of the Vα7.2^+^ T cells costained by the MR1-5RU tetramer for PBMCs vs. breast duct cells. **c** Flow cytometric analysis of CD161, interleukin (IL)-18Rα, and promyelocytic leukemia zinc finger protein (PLZF) staining for the subset of breast duct T cells that costain for Vα7.2 and MR1-5RU tetramer (*black line histogram*) compared with Vα7.2-negative T cells from the breast (*gray filled histogram*). *n.s.* Not significant

Breast tissue was obtained from human subjects who had undergone reduction mammoplasty or prophylactic mastectomy, and these tissues were subjected to a purification protocol that was optimized to isolate tissue fragments representing ductal organoids [10]. Purified ductal organoids were trypsinized to yield single-cell suspensions, and flow cytometric analysis was performed to identify T cells expressing Vα7.2 (Fig. 1a, left panel). Analysis of breast tissue samples from 16 unrelated donors revealed that the percentage of Vα7.2^+^ T cells varied over more than a 10-fold range, but the mean was nearly identical to that for Vα7.2^+^ T cells from blood of control donors (Fig. 1a, right panel). We next investigated staining by MR1 tetramers loaded with the 5RU antigen. For both PBMC and breast tissue samples, nearly 100% of the events stained by the MR1-5RU tetramer were positive for Vα7.2 (Fig. 1b, left panel). Neither PBMC nor breast duct organoid samples showed more than a marginal amount of staining using an MR1 tetramer loaded with a compound (6FP) that has been shown to have little or no antigenicity for MAIT cells (Additional file 2: Figure S1) [12, 26]. Surprisingly, whereas MR1-5RU tetramer-positive cells typically comprised at least half of the Vα7.2^+^ T cells in PBMCs, only a minority of the Vα7.2^+^ T cells from breast organoids were positively stained by the MR1-5RU tetramer (Fig. 1b, right panel). Nearly all of the Vα7.2^+^ MR1-5RU tetramer-positive cells from the breast duct were positive for CD161, and they also coexpressed IL-18Rα and PLZF (Fig. 1c). Together, these results establish that a population of Vα7.2^+^ T cells is present in human breast ducts that can be classified as MAIT cells, in that they bind MR1 molecules loaded with the 5RU antigen and coexpress CD161, IL-18Rα, and PLZF.

In contrast, the Vα7.2^+^ but MR1-5RU tetramer-negative population did not coexpress CD161, IL-18Rα, or PLZF (Additional file 3: Figure S2A), suggesting that they are not innate T lymphocytes. It remains unclear whether these Vα7.2^+^ T cells that are not stained by the MR1-5RU tetramer are simply not MR1-restricted T cells or whether they are MR1-restricted but fail to bind the 5RU-loaded tetramer because they recognize structurally distinct antigens. Notably, however, whereas most peripheral blood T cells are negative for CD69 and CD103 (αE integrin), nearly all of the T cells from breast duct (including both the MR1-5RU tetramer-positive and tetramer-negative populations) showed uniformly positive expression of CD69, and a large fraction coexpressed CD103 (Additional file 3: Figure S2B). Thus, the T cells associated with our breast duct organoid preparations had a phenotype similar to tissue-resident memory T-cell populations that have recently been described [27].

### TCR Vβ use

**Fig. 2:**
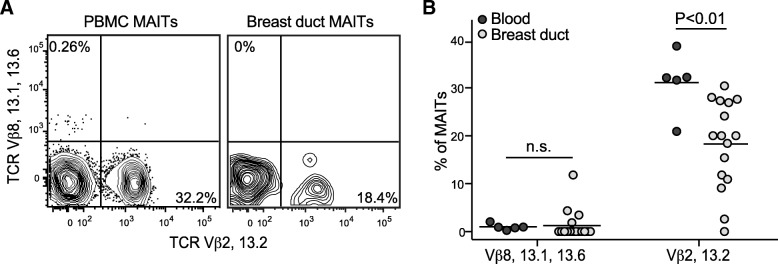
Breast duct mucosal associated invariant T (MAIT) cells show altered Vβ chain use frequencies compared with blood MAIT cells. **a** Flow cytometric staining to detect use of the indicated sets of Vβ chains by MAIT cells from blood (left panel) vs. breast duct (right panel). **b** Quantification of the fraction of MAIT cells staining positively for the indicated Vβ chains in peripheral blood mononuclear cells (PBMCs) or breast duct tissues obtained from female subjects. Each symbol represents the result from a different tissue donor. PBMCs and breast tissue samples were not obtained from the same donors. *TCR* T-cell receptor

Although the TCR α-chain sequences of MAIT cells are highly constrained (Vα7.2 paired with a limited set of Jα segments), their TCR β-chains are much more diverse and use a variety of different Vβ segments [22, 28, 29]. It has recently been shown that the specific TCR Vβ segments used by MAIT cells can influence their responsiveness to different bacterial species [30]. Because the microbial species encountered by breast duct MAIT cells likely differ at least in part from those encountered by MAIT cells circulating in blood, we investigated TCR Vβ use. Prior studies have established that the majority of MAIT cells in human blood express either Vβ2 or Vβ13.2, and MAIT cells with these Vβ chains are highly responsive to *E. coli* [30]. We found that, on average, less than 20% of the breast duct MR1-5RU tetramer-positive T cells expressed either of these two Vβ chains (Fig. 2a and b), and the frequency of breast duct MAIT cells expressing Vβ2 or Vβ13.2 appeared significantly lower than that for blood MAIT cells from a group of sex-matched control donors (Fig. 2b).

Conversely, MAIT cells using Vβ8, Vβ13.1, or Vβ13.6, which together made up only a minor fraction of the MAIT cells in blood, were previously found to have lower levels of responsiveness to *E. coli* [30]. There was little or no detectable expression of these Vβ segments by MAIT cells from most of the breast tissue samples we tested, although they appeared to be expanded in a few of the samples (Fig. 2b). Based on the low representation in the breast duct MAIT population of Vβ2 or Vβ13.2 TCRs, which are typically highly represented in the blood MAIT population, these results suggest that the breast duct MAIT cell population may have antigen specificity differences compared with those from blood.

### Cytokine production

**Fig. 3:**
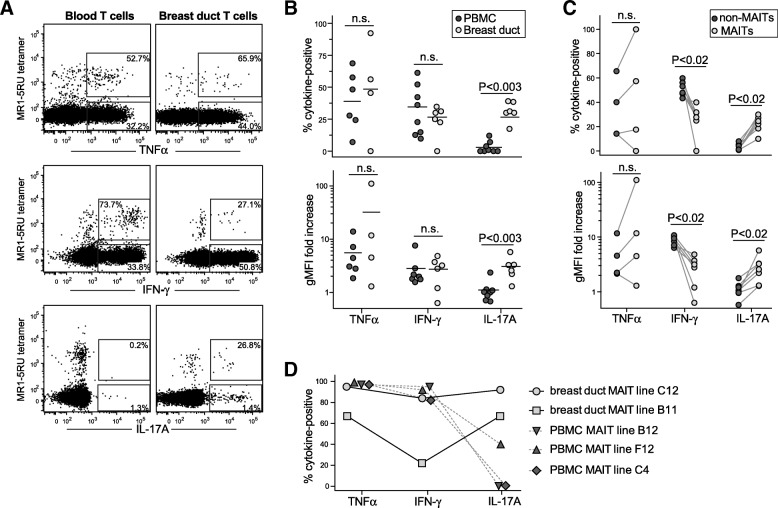
Mucosal associated invariant T (MAIT) cells from human breast ducts produce a more robust interleukin (IL)-17A response than blood MAIT cells. **a** Isolated breast duct cells or peripheral blood mononuclear cell (PBMC) samples were stimulated with phorbol 12-myristate 13-acetate (PMA) and ionomycin, then stained for expression of the indicated cytokines. Boxes show positive cytokine staining based on parallel analysis of unstimulated samples. Percentages shown within the gated areas represent the percentage of the total MAIT cells expressing cytokine (top boxes) or of the total non-MAIT cells (bottom boxes), respectively. **b** Top graph: Quantification of the fraction of MAIT cells from PBMCs (*dark circles*) vs. breast ducts (*light circles*) that stained positively for the indicated cytokines after PMA/ionomycin stimulation. Bottom graph: Fold increase in cytokine signal intensity. The geometric mean fluorescence intensity (gMFI) of the total MAIT cell population for the indicated cytokines was normalized by that of unstimulated MAIT cells. Each symbol represents the result from a different tissue donor; PBMC and breast tissue samples were not obtained from the same donors. **c** Top graph: Quantification of the fraction of MAIT cells (*light circles*) vs. non-MAIT cells (*dark circles*) from the same breast tissue sample that stained positively for the indicated cytokines after PMA/ionomycin stimulation. Bottom graph: Fold increase in cytokine signal intensity (gMFI of the total MAIT cell population normalized by that of unstimulated MAIT cells). Each symbol represents the result from a different breast tissue donor. **d** The indicated in vitro-expanded MAIT cell lines were stimulated with PMA and ionomycin, and expression of tumor necrosis factor (TNF)-α, interferon (IFN)-γ, and IL-17A was assessed by intracellular cytokine staining. The graph shows the percentage of MAIT cells in each line showing positive staining for the indicated cytokines (*see also* Additional file 4: Figure S3). *n.s.* Not significant; *5RU* 5-(2-oxoprophylideneamino)-6-d-ribitylaminouracil

We next compared the cytokine production profile of breast duct MAIT cells with those in PBMC samples. Total breast duct cells or PBMCs were stimulated with PMA and ionomycin, then stained them with MR1-5RU tetramer and anti-CD3 to detect MAIT cells and fixed, permeabilized, and stained them for expression of TNF-α, IFN-γ, and IL-17A. As also previously observed by others [20], blood MAIT cells produced almost exclusively IFN-γ and TNF-α (Fig. 3a, left panels). In contrast, the breast duct MAIT cells included cells producing TNF-α, IFN-γ, and IL-17A (Fig. 3a, right panels). Although the production of TNF-α and IFN-γ was not statistically different between MAIT cells from breast duct or blood, IL-17-producing MAIT cells were significantly enriched in breast duct compared with blood (Fig. 3b). Moreover, comparison of cytokine production by breast duct MAIT cells vs. the non-MAIT cells present in the breast duct organoid samples demonstrated significantly higher IL-17 production by the MAIT cell subset (Fig. 3c). These results demonstrate that, similar to what has recently been reported for the female genital tract [20], the breast duct MAIT cell population is enriched for IL-17 producers.

Costaining for IL-17A and IFN-γ indicated that most of the breast duct MAIT cells were single producers of these cytokines (data not shown). To further investigate, we generated short-term in vitro expansions of MAIT cells derived from breast ducts or blood. Five lines that each contained at least 95% MAIT cells as assessed by MR1 tetramer staining were selected for further analysis (two MAIT lines from breast duct tissue and three from blood). Using PMA/ionomycin stimulation to activate cytokine production, we observed that one of the breast duct lines (B11) showed mainly IL-17A single-positive cells (59%), along with 7.4% that were double-positive for IL-17/IFN-γ and 12% that were IFN-γ single-positive. The other breast-derived line (C12) showed mainly IL-17/IFN-γ double-positive cells (82%), along with 9% IL-17A single-positive cells and only very few (2.6%) IFN-γ single-positive cells. In contrast, two of the PBMC-derived MAIT lines (B12 and C4) contained nearly exclusively IFN-γ single-positive cells, whereas the third (F12) contained a majority (57%) of IFN-γ single-positive cells along with 39% that coexpressed IFN-γ and IL-17A (Fig. 3d and Additional file 4: Figure S3). These results suggest that MAIT cells which are stably polarized toward a Th17 phenotype are present in the breast duct compartment. Additionally, some of the breast duct MAIT cells may be Th1-polarized, and some may maintain plasticity in the ability to produce IFN-γ and IL-17.

### Activation of MAIT cells by breast carcinoma cells

**Fig. 4:**
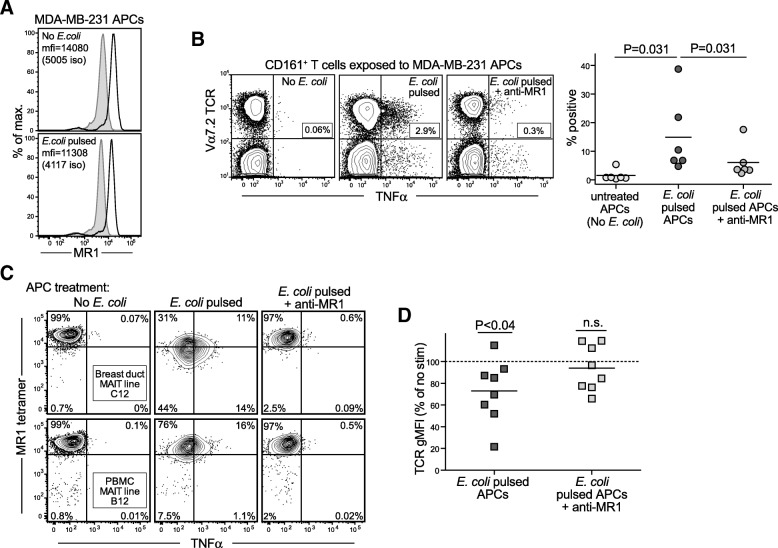
Breast epithelial carcinoma cells present microbial products to mucosal associated invariant T (MAIT) cells via MR1. **a** MDA-MB-231 breast carcinoma cells were exposed to killed *Escherichia coli* or mock-treated, then fixed and permeabilized and stained for MR1 (*black line*) compared with isotype control (*gray histogram*). **b** Left plots: Flow cytometric analysis showing tumor necrosis factor (TNF)-α production by primary human MAIT cells from peripheral blood in response to *E. coli*-treated MDA-MB-231 breast carcinoma cells. Cells expressing CD161 were magnetically sorted from freshly isolated peripheral blood mononuclear cells (PBMCs) to enrich for MAIT cells and to remove potential antigen-presenting cell (APC) populations. MDA-MB-231 cells were pulsed with fixed *E. coli* or mock-treated (no *E. coli*) and used to stimulate the CD161-selected population in the presence or absence of an anti-MR1 blocking antibody. Expression of TNF-α was measured by intracellular cytokine staining after ~ 24 hours. Graph on right: Quantification of the percentage of the MAIT cell population showing positive TNF-α staining in the indicated conditions. Each symbol represents the result of an independent analysis. **c** Intracellular cytokine staining showing TNF-α production by the indicated in vitro-expanded MAIT cells after exposure to mock-treated or *E. coli*-pulsed MDA-MB-231 cells in the presence or absence of an anti-MR1 blocking antibody. **d** T-cell receptor (TCR) cell surface staining of MAIT cells after exposure to *E. coli*-pulsed MDA-MB-231 cells in the presence or absence of an anti-MR1 blocking antibody. MAIT cell TCR expression (geometric mean fluorescence intensity [gMFI]) is expressed as a percentage of that for the corresponding MAIT cells exposed to mock-treated APCs. Each symbol represents the result of an independent analysis. Statistical results are for a two-tailed, one-sample *t* test comparison to a theoretical mean of 100%. *n.s.* Not significant

We next investigated the ability of human breast carcinoma cells to activate MAIT cells in an MR1-dependent manner. Prior researchers have observed MR1 gene expression by a variety of different cell types, including neoplastic cells of epithelial origin, such as HeLa cells (cervical carcinoma) and A549 (lung carcinoma) [31, 32]. However, it has typically been difficult to detect cell surface expression of endogenous MR1 molecules by flow cytometry, likely because antigen binding is strictly required for export of MR1 molecules to the cell surface and because MR1 expression at the cell surface is highly transient [33, 34]. We therefore used intracellular flow cytometric staining to confirm that the human breast carcinoma cell line MDA-MB-231 endogenously expresses the MR1 antigen-presenting molecule (Fig. 4a).

Although MAIT cells can produce IFN-γ in a TCR-independent manner in response to IL-18 exposure [35, 36], it has recently been shown that they produce TNF-α selectively as a result of TCR-mediated activation [30]. Therefore, we investigated the ability of primary human MAIT cells to produce TNF-α in response to MDA-MB-231 breast carcinoma cells. Freshly isolated PBMCs were enriched for CD161^+^ cells by magnetic sorting, yielding a preparation that contained almost exclusively CD161^+^ T cells and natural killer (NK) cells (Additional file 1: Figure S4). MDA-MB-231 breast carcinoma cells were exposed to killed *E. coli* bacteria or mock-treated, then washed and coincubated with the isolated CD161^+^ cells in the presence or absence of an anti-MR1 blocking antibody. There was little evidence of TNF-α production by MAIT cells in response to the mock-treated breast carcinoma lines, whereas the *E. coli*-treated cells elicited a robust TNF-α response (Fig. 4b). MAIT cell cytokine production was markedly reduced in the presence of the anti-MR1 blocking antibody (Fig. 4b), indicating that the *E. coli*-dependent response was due to recognition of MR1. We also observed similar MR1-dependent TNF-α production in response to *E. coli*-pulsed MDA-MB-231 cells by our in vitro expanded MAIT lines (Fig. 4c). These results suggested that the MDA-MB-231 cells are able to take up bacterially derived extracellular antigens and present them to MAIT cells via MR1.

Further supporting a role for TCR recognition in MAIT cell responses to the MDA-MB-231 cells, we noted that the responding MAIT cells showed somewhat reduced staining by reagents specific for MAIT TCRs (i.e., either Vα7.2 monoclonal antibody or MR1 tetramer; *see* flow cytometric analyses shown in Fig. 4b and c). It is now well established that antigenic activation of T cells results in increased internalization of TCR-CD3 complexes and thus leads to reduced cell surface expression levels of these molecules [37]. MAIT cells that were exposed to *E. coli*-pulsed MDA-MB-231 cells reproducibly showed reduced TCR cell surface expression compared with MAIT cells that were exposed to mock-treated MDA-MB-231 cells (Fig. 4d). Moreover, the TCR downregulation was prevented in the presence of an anti-MR1 blocking antibody (Fig. 4d). Together, these results demonstrate that MAIT cells are activated in a TCR-, MR1-, and microbe-dependent manner by MDA-MB-231 breast carcinoma cells.

### Breast carcinoma cells elicit an IL-17-skewed response by breast duct MAIT cells

**Fig. 5:**
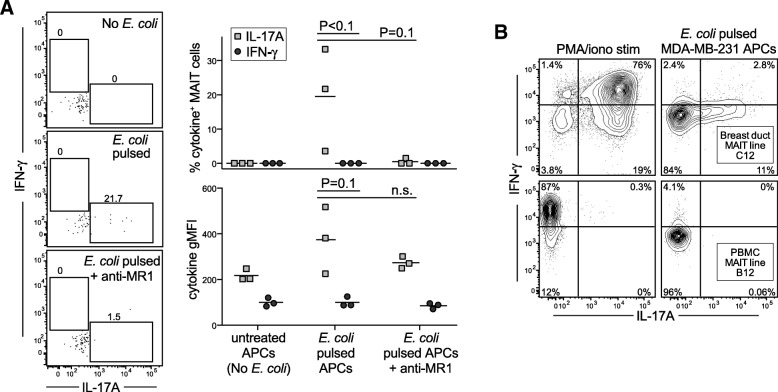
**a** Cells isolated from primary human breast ducts were exposed to MDA-MB-231 breast carcinoma cells that were mock-treated or pulsed with fixed *Escherichia coli* in the presence or absence of an anti-MR1 blocking antibody. Left plots: Flow cytometric analysis of intracellular interferon (IFN)-γ and interleukin (IL)-17A staining by the mucosal associated invariant T (MAIT) cell population from one representative experiment. Graphs on right: Intracellular cytokine analysis results from three independent experiments. **b** In vitro-expanded MAIT cells derived from breast duct (top row) or from peripheral blood mononuclear cells (PBMCs) (bottom row) were stimulated with phorbol 12-myristate 13-acetate (PMA) and ionomycin (left column) or exposed to *E. coli*-pulsed MDA-MB-231 cells (right column). Expression of IFN-γ and IL-17A was assessed by intracellular cytokine staining. *APCs* Antigen-presenting cells

On the basis of our analyses of MAIT cell responses to PMA/ionomycin stimulation (Fig. 3a–c), it was clear that the breast duct MAIT population includes both IFN-γ and IL-17A producers. We were therefore surprised to observe that primary breast duct MAIT cells showed production of IL-17A but no detectable IFN-γ in response to *E. coli*-pulsed MDA-MB-231 cells (Fig. 5a). To further investigate, we tested the responses of two in vitro expanded MAIT lines to *E. coli*-pulsed MDA-MB-231 cells. Whereas most MAIT cells within the C12 breast duct-derived MAIT line produced both IFN-γ and IL-17A in response to PMA/ionomycin stimulation, their response to the breast carcinoma cells was nearly exclusively limited to IL-17A production (Fig. 5b, top panels). The PBMC-derived MAIT line B12, which produced IFN-γ but not IL-17A in response to PMA/ionomycin, showed only modest IFN-γ secretion in response to *E. coli*-pulsed MDA-MB-231 cells and no detectable IL-17A (Fig. 5b, bottom panels). Addition of an anti-MR1 blocking antibody nearly completely prevented these cytokine responses to the *E. coli*-pulsed APCs, and neither MAIT cell line showed detectable responses to mock-treated MDA-MB-231 cells (data not shown). Thus, these results reveal a surprising bias toward IL-17A production by breast duct MAIT cells in response to bacterially treated breast carcinoma cells.

### Role of NKG2D in MAIT cell responses to breast carcinoma cells

**Fig. 6:**
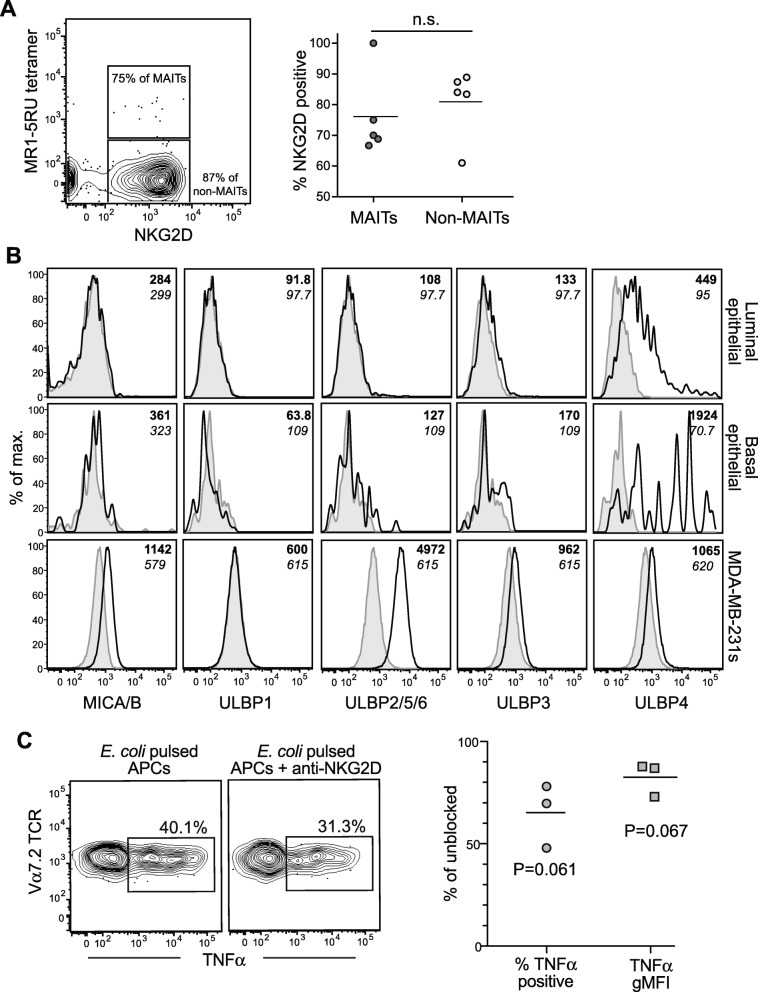
Natural killer group 2 member D (NKG2D) plays a role in mucosal associated invariant T (MAIT) cell activation by breast carcinoma cells. **a** Left plot: Flow cytometric analysis showing NKG2D receptor expression by primary MAIT cells in a breast duct tissue sample. Right graph: Aggregated results from five different breast tissue samples showing the percentage of MAIT cells or non-MAIT cells staining positively for NKG2D. **b** Flow cytometric analysis of the expression of known NKG2D ligands (major histocompatibility complex class I-related chain A and B [MICA/B], UL16-binding proteins 1–6 [ULBP1–6]) by primary nontransformed breast duct epithelial cells (top row, luminal subset; middle row, basal subset) compared with MDA-MB-231 breast carcinoma cells (bottom row). Staining with specific antibodies is shown as an *open histogram* with a *heavy black line* with geometric mean fluorescence intensity (gMFI) indicated in bold; *gray filled histograms* show isotype-matched negative control antibody staining with gMFI indicated in italic font. **c** Left plots: Tumor necrosis factor (TNF)-α expression by primary MAIT cells in CD161-selected peripheral blood mononuclear cell samples in response to *Escherichia coli*-pulsed MDA-MB-231 breast carcinoma cells in the presence or absence of an anti-NKG2D blocking antibody. Right graph: Quantification of anti-NKG2D blocking effect. The percentage of MAIT cells showing positive cytokine expression in the NKG2D blockade condition was normalized by the percentage positive in the unblocked condition. Symbols represent the results of independent experiments. *P* values are derived from two-tailed, one-sample *t* tests. *APCs* Antigen-presenting cells, *n.s.* Not significant, *5RU* 5-(2-oxoprophylideneamino)-6-d-ribitylaminouracil

Prior analyses have revealed that peripheral blood MAIT cells are positive for NKG2D, a receptor that recognizes a series of ligands that become upregulated on stressed and neoplastic cells [14]. We found that most MAIT cells from breast ducts show intermediate to high levels of NKG2D expression, although most non-MAIT cells from breast ducts also expressed NKG2D (Fig. 6a). Comparing NKG2D counterligand expression by primary breast duct epithelial cells with that of MDA-MB-231 carcinoma cells, we found that primary epithelial cells of either a luminal or basal phenotype lacked detectable expression of MICA/B or ULBP isoform 1, 2, 3, 5, or 6 (Fig. 6b). However, there was detectable expression of ULBP4 on both luminal and basal epithelial cells (Fig. 6b). In contrast, MDA-MB-231 cells showed positive staining for MICA/B and also for some of the ULBP isoforms (Fig. 6b). MAIT cells that were exposed to *E. coli*-pulsed MDA-MB-231 cells in the presence of an anti-NKG2D blocking antibody consistently showed a partial reduction in TNF-α production (Fig. 6c). These results suggest that, though not the dominant pathway of activation, NKG2D-mediated stimulation may costimulate the TCR-mediated activation of MAIT cells by *E. coli-*pulsed breast carcinoma cells.

## Discussion

We show in the present study that MAIT cells are present in the epithelial ducts of human breast tissue and that MAIT cells mediate effector responses to breast carcinoma cells that have been exposed to microbial compounds. The role that the breast duct microbiome plays in breast cancer initiation and progression is currently completely uncharted, because the composition and dynamics of the breast duct microbiota are just beginning to be explored. However, intriguing hints are starting to emerge that dysbiosis of the normal breast duct microbiota may be associated with breast cancer, because recent studies have documented differences in the composition of the microbial taxa associated with breast cancer tissue compared with healthy breast tissue [2–4, 6–8]. Given that MAIT cells specifically recognize riboflavin metabolites as antigens presented by MR1 molecules, it is of particular interest that riboflavin-producing bacterial species have been found to be present at higher relative abundance in women with breast cancer, including *Enterobacteriaceae* (the family that includes *E. coli*), *Bacillus*, and *Staphylococcus* species [5, 6]. Thus, understanding the phenotypic and functional properties of the MAIT cells found within breast ducts is an important step in determining whether these cells play a role during the carcinogenesis of breast duct epithelial cells.

In contrast to MAIT cells from peripheral blood, which show almost exclusively a Th1 phenotype, we observed that about one-third of the breast duct MAIT cell population produced IL-17A. Similarly, MAIT cells from the female genital tract were recently shown to be enriched for IL-17A and IL-22 production [20]. Hence, Th17 polarization may be a common feature of MAIT cells from ductal and skin epithelial tissues. Because IL-17A enhances epithelial barrier integrity, MAIT cells with Th17 polarization might contribute to the maintenance of a healthy epithelial surface. It is not clear whether secretion of IL-17A promotes or hinders the initial stages of epithelial carcinogenesis. However, there is a growing body of evidence that after tumors are established, IL-17A has protumorigenic functions, because IL-17A has been shown to be produced by breast cancer tumor-infiltrating lymphocytes and thereby to promote chemoresistance, proliferation, and migration of breast cancer cells [38]. Thus, it will be of great interest to determine whether IL-17A-producing MAIT cells are selectively expanded within breast carcinoma tissue, as has been observed recently for colon carcinomas [24].

In contrast, the IFN-γ-producing subset of breast duct MAIT cells might be expected to have antitumor functions. However, we found that breast MAIT cells that were exposed to *E. coli*-pulsed breast carcinoma cells produced only IL-17A and TNF-α. It is not clear whether the breast carcinoma cells somehow selectively suppress MAIT cell IFN-γ production. Alternatively, it is possible that cancerous cells that have taken up enterobacterial antigens selectively activate only the Th17-polarized subset of breast duct MAIT cells. One mechanism that might explain the selective activation of a particular functional subset of MAIT cells is the observation that the Vβ chain use of the TCR influences the ability of MAIT cells to respond to microbial species, presumably as a result of structural or quantitative variations in the antigenic compounds produced by different types of bacteria [30]. We found that the breast duct MAIT cell population appeared to have a comparatively low frequency of TCRs that use the two Vβ chains that are most common in the blood MAIT cell population (i.e., Vβ2 and Vβ13.2). Because these Vβ chains have been shown to confer stronger reactivity to *E. coli* [30], this observation would be consistent with the possibility that the TCR repertoire of the breast duct MAIT cell population is shaped by a distinct composition of microbial colonists. If this is the case, an intriguing extension of this hypothesis is the possibility that specific functional subsets of MAIT cells (e.g., Th1- vs. Th17-polarized) bear distinct TCR Vβ uses and thus differ in their microbial reactivity.

It is also interesting that in contrast to the blood, where more than half of the Vα7.2^+^ T cells typically are stained by the MR1-5RU tetramer, only about one-third of the breast duct Vα7.2^+^ T cells were tetramer-positive. One potential explanation for this is that the tetramer-negative Vα7.2^+^ T cells recognize completely distinct antigens presented by MR1 and therefore are not bound by MR1 tetramers loaded with the 5RU compound. Alternatively, these cells may simply not be MR1-restricted. If this is the case, it is intriguing to speculate that they may belong to another conserved Vα7.2^+^ T-cell population that is now known as germline-encoded mycolyl-reactive T cells. This subset of T cells also uses Vα7.2 and has been shown to be restricted by CD1b and to recognize mycolate antigens produced by mycobacterial species [39].

A further question of interest relates to the features of breast duct epithelial cells that are required to activate the responses of breast duct MAIT cells. We found that MAIT cells are readily activated by breast carcinoma cells and that this activation is dependent on prior bacterial exposure of the carcinoma cells and can be blocked by an anti-MR1 antibody. It is not yet clear whether normal (i.e., nontransformed) breast duct epithelial cells are also able to activate MAIT cells via MR1-mediated antigen presentation or whether their activation is normally mediated by other immune cell types present in the local tissues (e.g., monocytes, macrophages, or dendritic cells). However, because neoplastic cells often upregulate ligands for NKG2D, and because the addition of an NKG2D blocking antibody resulted in a partial reduction of MAIT cell effector responses, it is possible that the NKG2D pathway promotes the ability of MAIT cells to detect and respond to breast carcinomas.

## Conclusions

Together, these findings underscore the likelihood that there exists a tripartite interaction among MAIT cells, breast duct epithelial cells, and the breast microbiome that may play a role in breast carcinogenesis or during the progression of established tumors. Because breast duct MAIT cells appear to use distinct TCR sequences and include multiple functional subsets (e.g., Th1- or Th17-polarized), the breast-resident MAIT cell population likely mediates divergent responses to different types of challenge. Our data suggest that microbially exposed breast carcinoma cells may selectively activate only Th17-polarized MAIT cells from breast ducts and not the Th1-polarized subset. Therefore, a critical question for future analysis is to investigate whether Th17-polarized MAIT cells are enriched in breast carcinomas and how this impacts prognosis.

## Additional files

Additional file 1: **Figure S4.** Magnetic sorting of CD161^+^ cells from PBMCs yields enrichment of MAIT cells and depletion of nonlymphocytic antigen-presenting cells. Flow cytometric analysis of the CD161-enriched fraction, showing the MAIT cell population (far right plot) and demonstrating that the CD3^−^ population is almost entirely comprised of NK cells as assessed by NKp46 and CD56 expression. (PDF 235 kb)

Additional file 2: **Figure S1.** Validation of MR1 tetramer staining. MR1-5RU tetramer staining (1:100 dilution) and MR1-6FP tetramer (1:100 dilution) in combination with Vα7.2 on CD3^+^ cells from (**a**) PBMCs and (**b**) breast ducts. (PDF 401 kb)

Additional file 3: **Figure S2.** Phenotypic analyses of breast duct lymphocytes. **a** CD161, IL-18Rα, and PLZF expression on Vα7.2^+^ T cells that do *not* costain with MR1-5RU tetramer (*black line*) compared with Vα7.2^−^CD3^+^ T cells (*filled gray histogram*). **b** In contrast to most T cells from PBMCs, T lymphocytes from breast ducts show a tissue-resident memory phenotype (CD69^+^ and CD103^+^). (PDF 270 kb)

Additional file 4: **Figure S3.** IFN-γ vs. IL-17A production by in vitro-expanded MAIT lines after PMA/ionomycin stimulation. In vitro-expanded MAIT cells derived from breast duct (left column) or from PBMCs (right column) were stimulated with PMA and ionomycin, and expression of IFN-γ and IL-17A was assessed by intracellular cytokine staining. (PDF 252 kb)

## References

[1] Visvader JE (2009). Keeping abreast of the mammary epithelial hierarchy and breast tumorigenesis. Genes Dev.

[2] Chan AA, Bashir M, Rivas MN, Duvall K, Sieling PA, Pieber TR, Vaishampayan PA, Love SM, Lee DJ (2016). Characterization of the microbiome of nipple aspirate fluid of breast cancer survivors. Sci Rep.

[3] Hieken TJ, Chen J, Hoskin TL, Walther-Antonio M, Johnson S, Ramaker S, Xiao J, Radisky DC, Knutson KL, Kalari KR (2016). The microbiome of aseptically collected human breast tissue in benign and malignant disease. Sci Rep.

[4] Thompson KJ, Ingle JN, Tang X, Chia N, Jeraldo PR, Walther-Antonio MR, Kandimalla KK, Johnson S, Yao JZ, Harrington SC (2017). A comprehensive analysis of breast cancer microbiota and host gene expression. PLoS One.

[5] Urbaniak C, Cummins J, Brackstone M, Macklaim JM, Gloor GB, Baban CK, Scott L, O’Hanlon DM, Burton JP, Francis KP (2014). Microbiota of human breast tissue. Appl Environ Microbiol.

[6] Urbaniak C, Gloor GB, Brackstone M, Scott L, Tangney M, Reid G (2016). The microbiota of breast tissue and its association with breast cancer. Appl Environ Microbiol.

[7] Wang H, Altemus J, Niazi F, Green H, Calhoun BC, Sturgis C, Grobmyer SR, Eng C (2017). Breast tissue, oral and urinary microbiomes in breast cancer. Oncotarget.

[8] Xuan C, Shamonki JM, Chung A, Dinome ML, Chung M, Sieling PA, Lee DJ (2014). Microbial dysbiosis is associated with human breast cancer. PLoS One.

[9] Russo E, Taddei A, Ringressi MN, Ricci F, Amedei A (2016). The interplay between the microbiome and the adaptive immune response in cancer development. Therap Adv Gastroenterol.

[10] Zumwalde NA, Haag JD, Sharma D, Mirrielees JA, Wilke LG, Gould MN, Gumperz JE (2016). Analysis of immune cells from human mammary ductal epithelial organoids reveals Vδ2^+^ T cells that efficiently target breast carcinoma cells in the presence of bisphosphonate. Cancer Prev Res (Phila).

[11] Treiner E, Duban L, Bahram S, Radosavljevic M, Wanner V, Tilloy F, Affaticati P, Gilfillan S, Lantz O (2003). Selection of evolutionarily conserved mucosal-associated invariant T cells by MR1. Nature.

[12] Kjer-Nielsen L, Patel O, Corbett AJ, Le Nours J, Meehan B, Liu L, Bhati M, Chen Z, Kostenko L, Reantragoon R (2012). MR1 presents microbial vitamin B metabolites to MAIT cells. Nature.

[13] Reantragoon R, Kjer-Nielsen L, Patel O, Chen Z, Illing PT, Bhati M, Kostenko L, Bharadwaj M, Meehan B, Hansen TH (2012). Structural insight into MR1-mediated recognition of the mucosal associated invariant T cell receptor. J Exp Med.

[14] Dusseaux M, Martin E, Serriari N, Peguillet I, Premel V, Louis D, Milder M, Le Bourhis L, Soudais C, Treiner E (2011). Human MAIT cells are xenobiotic-resistant, tissue-targeted, CD161hi IL-17-secreting T cells. Blood.

[15] Savage AK, Constantinides MG, Han J, Picard D, Martin E, Li B, Lantz O, Bendelac A (2008). The transcription factor PLZF directs the effector program of the NKT cell lineage. Immunity.

[16] Reantragoon R, Corbett AJ, Sakala IG, Gherardin NA, Furness JB, Chen Z, Eckle SB, Uldrich AP, Birkinshaw RW, Patel O (2013). Antigen-loaded MR1 tetramers define T cell receptor heterogeneity in mucosal-associated invariant T cells. J Exp Med.

[17] Gold MC, Cerri S, Smyk-Pearson S, Cansler ME, Vogt TM, Delepine J, Winata E, Swarbrick GM, Chua WJ, Yu YY (2010). Human mucosal associated invariant T cells detect bacterially infected cells. PLoS Biol.

[18] Peterfalvi A, Gomori E, Magyarlaki T, Pal J, Banati M, Javorhazy A, Szekeres-Bartho J, Szereday L, Illes Z (2008). Invariant Vα7.2-Jα33 TCR is expressed in human kidney and brain tumors indicating infiltration by mucosal-associated invariant T (MAIT) cells. Int Immunol.

[19] Serriari NE, Eoche M, Lamotte L, Lion J, Fumery M, Marcelo P, Chatelain D, Barre A, Nguyen-Khac E, Lantz O (2014). Innate mucosal-associated invariant T (MAIT) cells are activated in inflammatory bowel diseases. Clin Exp Immunol.

[20] Gibbs A, Leeansyah E, Introini A, Paquin-Proulx D, Hasselrot K, Andersson E, Broliden K, Sandberg JK, Tjernlund A (2017). MAIT cells reside in the female genital mucosa and are biased towards IL-17 and IL-22 production in response to bacterial stimulation. Mucosal Immunol.

[21] Leeansyah E, Loh L, Nixon DF, Sandberg JK (2014). Acquisition of innate-like microbial reactivity in mucosal tissues during human fetal MAIT-cell development. Nat Commun.

[22] Lepore M, Kalinichenko A, Colone A, Paleja B, Singhal A, Tschumi A, Lee B, Poidinger M, Zolezzi F, Quagliata L (2014). Parallel T-cell cloning and deep sequencing of human MAIT cells reveal stable oligoclonal TCRβ repertoire. Nat Commun.

[23] Dias J, Sobkowiak MJ, Sandberg JK, Leeansyah E (2016). Human MAIT-cell responses to *Escherichia coli*: activation, cytokine production, proliferation, and cytotoxicity. J Leukoc Biol..

[24] Sundstrom P, Ahlmanner F, Akeus P, Sundquist M, Alsen S, Yrlid U, Borjesson L, Sjoling A, Gustavsson B, Wong SB (2015). Human mucosa-associated invariant T cells accumulate in colon adenocarcinomas but produce reduced amounts of IFN-γ. J Immunol.

[25] Ling L, Lin Y, Zheng W, Hong S, Tang X, Zhao P, Li M, Ni J, Li C, Wang L (2016). Circulating and tumor-infiltrating mucosal associated invariant T (MAIT) cells in colorectal cancer patients. Sci Rep.

[26] Patel O, Kjer-Nielsen L, Le Nours J, Eckle SB, Birkinshaw R, Beddoe T, Corbett AJ, Liu L, Miles JJ, Meehan B (2013). Recognition of vitamin B metabolites by mucosal-associated invariant T cells. Nat Commun.

[27] Kumar BV, Ma W, Miron M, Granot T, Guyer RS, Carpenter DJ, Senda T, Sun X, Ho SH, Lerner H (2017). Human tissue-resident memory T cells are defined by Core transcriptional and functional signatures in lymphoid and mucosal sites. Cell Rep.

[28] Gold MC, McLaren JE, Reistetter JA, Smyk-Pearson S, Ladell K, Swarbrick GM, Yu YY, Hansen TH, Lund O, Nielsen M (2014). MR1-restricted MAIT cells display ligand discrimination and pathogen selectivity through distinct T cell receptor usage. J Exp Med.

[29] Eckle SB, Birkinshaw RW, Kostenko L, Corbett AJ, McWilliam HE, Reantragoon R, Chen Z, Gherardin NA, Beddoe T, Liu L (2014). A molecular basis underpinning the T cell receptor heterogeneity of mucosal-associated invariant T cells. J Exp Med.

[30] Dias J, Leeansyah E, Sandberg JK (2017). Multiple layers of heterogeneity and subset diversity in human MAIT cell responses to distinct microorganisms and to innate cytokines. Proc Natl Acad Sci U S A.

[31] Laugel B, Lloyd A, Meermeier EW, Crowther MD, Connor TR, Dolton G, Miles JJ, Burrows SR, Gold MC, Lewinsohn DM (2016). Engineering of isogenic cells deficient for MR1 with a CRISPR/Cas9 lentiviral system: tools to study microbial antigen processing and presentation to human MR1-restricted T cells. J Immunol.

[32] Riegert P, Wanner V, Bahram S (1998). Genomics, isoforms, expression, and phylogeny of the MHC class I-related MR1 gene. J Immunol.

[33] Chua WJ, Kim S, Myers N, Huang S, Yu L, Fremont DH, Diamond MS, Hansen TH (2011). Endogenous MHC-related protein 1 is transiently expressed on the plasma membrane in a conformation that activates mucosal-associated invariant T cells. J Immunol.

[34] McWilliam HE, Eckle SB, Theodossis A, Liu L, Chen Z, Wubben JM, Fairlie DP, Strugnell RA, Mintern JD, McCluskey J (2016). The intracellular pathway for the presentation of vitamin B-related antigens by the antigen-presenting molecule MR1. Nat Immunol.

[35] Loh L, Wang Z, Sant S, Koutsakos M, Jegaskanda S, Corbett AJ, Liu L, Fairlie DP, Crowe J, Rossjohn J (2016). Human mucosal-associated invariant T cells contribute to antiviral influenza immunity via IL-18-dependent activation. Proc Natl Acad Sci U S A.

[36] Ussher JE, Bilton M, Attwod E, Shadwell J, Richardson R, de Lara C, Mettke E, Kurioka A, Hansen TH, Klenerman P (2014). CD161^++^CD8^+^T cells, including the MAIT cell subset, are specifically activated by IL-12+IL-18 in a TCR-independent manner. Eur J Immunol.

[37] Alcover A, Alarcon B (2000). Internalization and intracellular fate of TCR-CD3 complexes. Crit Rev Immunol.

[38] Cochaud S, Giustiniani J, Thomas C, Laprevotte E, Garbar C, Savoye AM, Cure H, Mascaux C, Alberici G, Bonnefoy N (2013). IL-17A is produced by breast cancer TILs and promotes chemoresistance and proliferation through ERK1/2. Sci Rep.

[39] Van Rhijn I, Kasmar A, de Jong A, Gras S, Bhati M, Doorenspleet ME, de Vries N, Godfrey DI, Altman JD, de Jager W (2013). A conserved human T cell population targets mycobacterial antigens presented by CD1b. Nat Immunol.

