# Supplementary figures and images for: Mucosal associated invariant T cells from human breast ducts mediate a Th17-skewed response to bacterially exposed breast carcinoma cells
Source: Breast Cancer Res. 2018 Sep 12;20:111. doi: 10.1186/s13058-018-1036-5 (PMC6134631; doi:10.1186/s13058-018-1036-5)

**Figure S4**

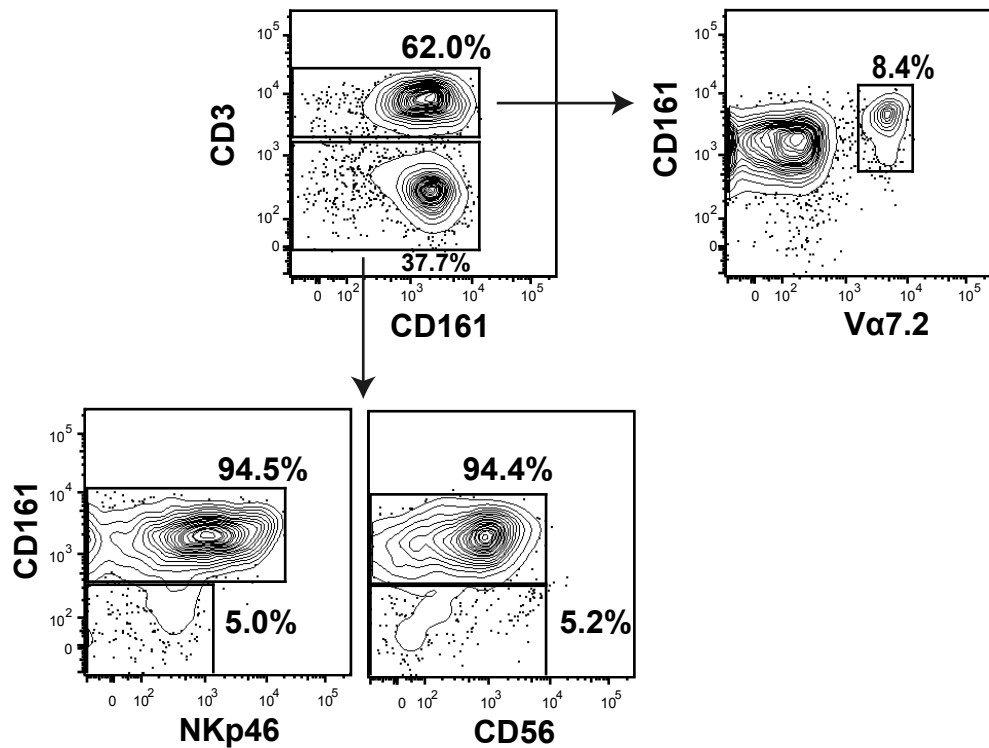

Supplement: Supplementary file 1 — Figure S4. Magnetic sorting of CD161+ cells from PBMCs yields enrichment of MAIT cells and depletion of nonlymphocytic antigen-presenting cells. Flow cytometric analysis of the CD161-enriched fraction, showing the MAIT cell population (far right plot) and demonstrating that the CD3− population is almost entirely comprised of NK cells as assessed by NKp46 and CD56 expression. (PDF 235 kb) [file 13058_2018_1036_MOESM1_ESM.pdf]

# Figure S1

## A

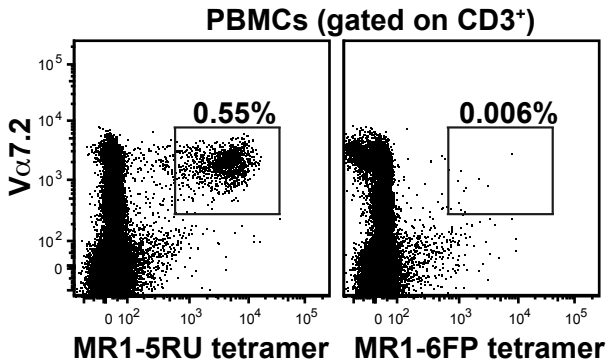

## B

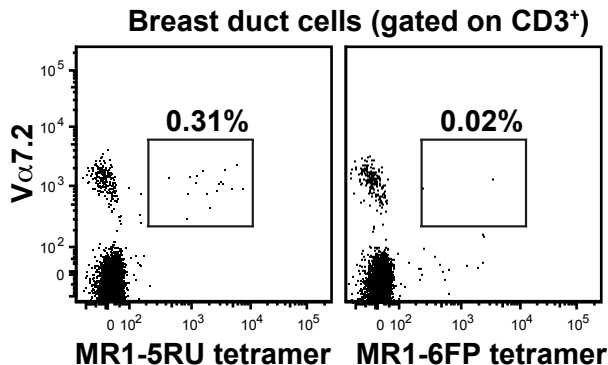

Supplement: Supplementary file 2 — Figure S1. Validation of MR1 tetramer staining. MR1-5RU tetramer staining (1:100 dilution) and MR1-6FP tetramer (1:100 dilution) in combination with Vα7.2 on CD3+ cells from (a) PBMCs and (b) breast ducts. (PDF 401 kb) [file 13058_2018_1036_MOESM2_ESM.pdf]

Figure S2

A

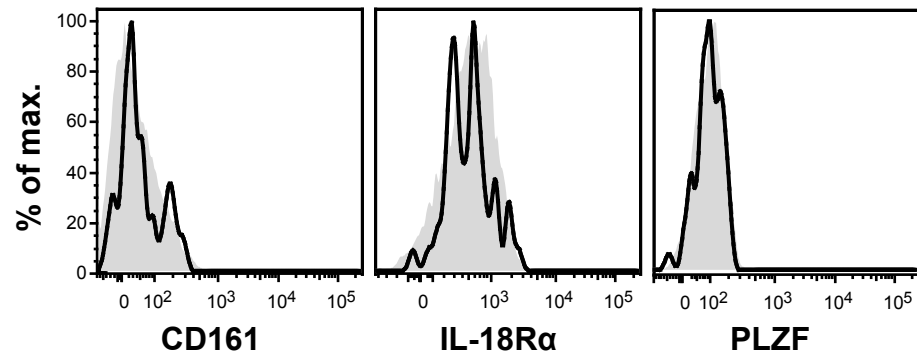

B

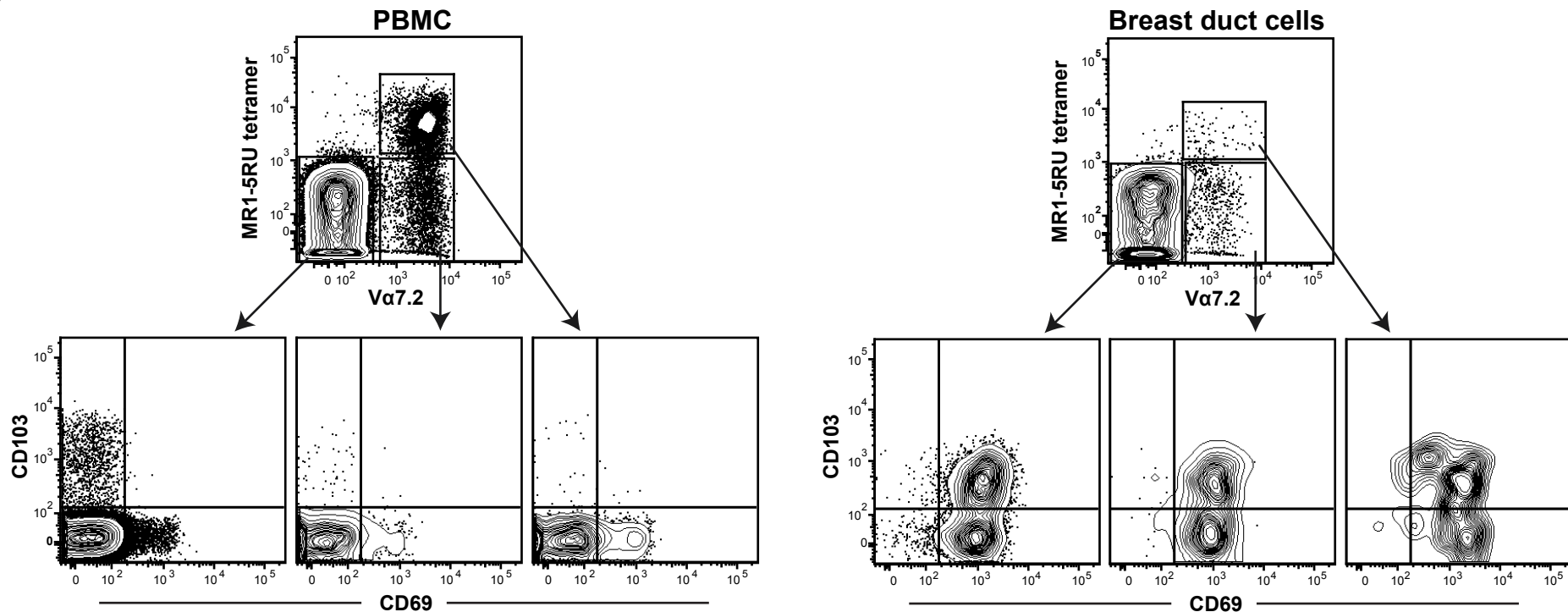

Supplement: Supplementary file 3 — Figure S2. Phenotypic analyses of breast duct lymphocytes. a CD161, IL-18Rα, and PLZF expression on Vα7.2+ T cells that do not costain with MR1-5RU tetramer (black line) compared with Vα7.2−CD3+ T cells (filled gray histogram). b In contrast to most T cells from PBMCs, T lymphocytes from breast ducts show a tissue-resident memory phenotype (CD69+ and CD103+). (PDF 270 kb) [file 13058_2018_1036_MOESM3_ESM.pdf]

# Figure S3

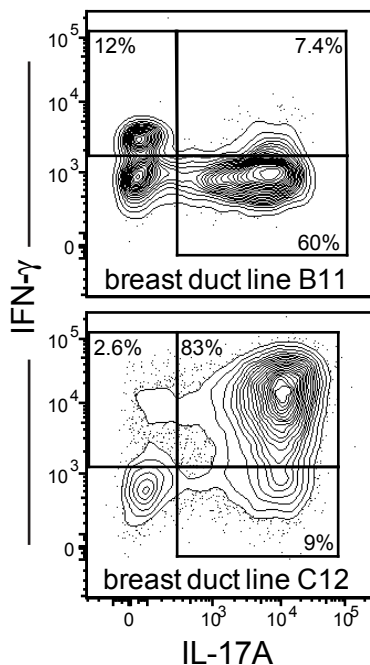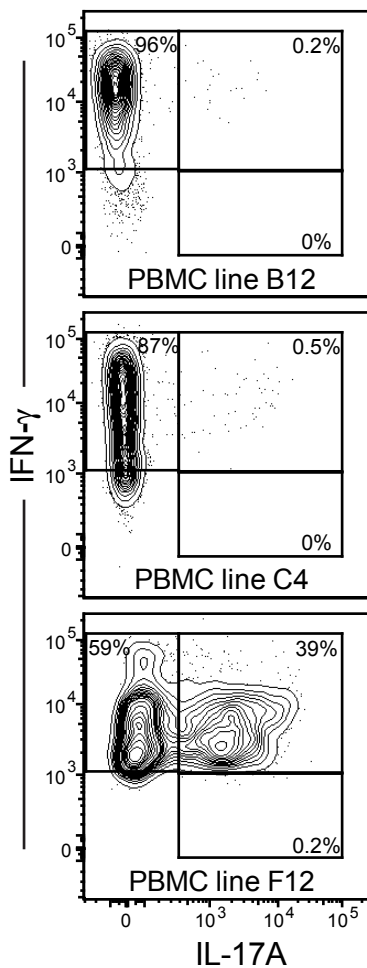

Supplement: Supplementary file 4 — Figure S3. IFN-γ vs. IL-17A production by in vitro-expanded MAIT lines after PMA/ionomycin stimulation. In vitro-expanded MAIT cells derived from breast duct (left column) or from PBMCs (right column) were stimulated with PMA and ionomycin, and expression of IFN-γ and IL-17A was assessed by intracellular cytokine staining. (PDF 252 kb) [file 13058_2018_1036_MOESM4_ESM.pdf]
